# Benzodiazepines prescribing in anxiety : Between practice and guidelines

**DOI:** 10.1192/j.eurpsy.2021.1294

**Published:** 2021-08-13

**Authors:** M. Lagha, U. Ouali, F. Nacef

**Affiliations:** Department Of Psychiatry A, Razi hospital, Manouba, Tunisia

**Keywords:** Benzodiazepines, guidelines, Prescribing, Anxiety

## Abstract

**Introduction:**

Benzodiazepines (BZD) are psychotropic drugs prescribed in psychiatry for their anxiolytic, hypnotic and sedative properties. Several guidelines aimed to limit the chronic use of BZDs. However, BZDs prescribing that does not comply with international recommendations remains widespread, estimated in France at 30% for anxiolytic BZDs.

**Objectives:**

The aims of our study were to evaluate BZDs prescribing practices in the treatment of anxiety and to assess their compliance with international recommendations.

**Methods:**

This is a cross-sectional study conducted through a Google-forms self-administered questionnaire,intended for psychiatrists and psychiatric residents, over a period of two months, from April 1 to May 31, 2019.

**Results:**

One hundred physicians practicing in psychiatry answered our questionnaire. The response rate was 28%. The most prescribed BZD for anxiolytic purposes was Prazepam (76.2%). Clonazepam was prescribed for anxiolytic purposes in 10.5% of cases. Of the 105 participants, 48 indicated that they prescribed BZDs for anxiolytic purposes in states of acute stress (45.7%), 28.6% prescribed them for the treatment of mild to moderate anxiety manifestations in anxiety disorders. For the treatment of anxiety without panic attacks, 20% indicated that they prefer a short half-life BZD, 80% a long half-life BZD. The maximum duration of BZDs prescription for anxiolytic purposes was 12 weeks (62%), and 6 months in 10% of cases.

**Conclusions:**

BZDs are often prescribed in psychiatry for their anxiolytic property, sometimes in a way that does not comply with the recommendations of good practice, with regard to the prescribed molecules, their indications and the duration and modalities of prescription.

